# Dialysis modality and survival in octogenarians: real-world population-based cohort study

**DOI:** 10.1093/ckj/sfag233

**Published:** 2026-07-15

**Authors:** Néstor Toapanta, María Antonieta Azancot, Jordi Comas, Natalia Ramos, Juan León-Román, Marc Patricio-Liébana, Jorge Sánchez Olaya, Tiffany Alvarez, Efraín Tatis, Sara Nuñez, Natalia Padron, Héctor Bedoya, Iván Zamora, Jaume Tort, Oriol Bestard, Francesc Moreso, María José Soler

**Affiliations:** Nephrology Department, Vall d’Hebron University Hospital, Vall d’Hebron Research Institute (VHIR), Vall d’Hebron Barcelona Hospital Campus, Autonomous University of Barcelona, Barcelona, Spain; Nephrology Department, Vall d’Hebron University Hospital, Vall d’Hebron Research Institute (VHIR), Vall d’Hebron Barcelona Hospital Campus, Autonomous University of Barcelona, Barcelona, Spain; Catalan Transplantation Organization, Barcelona, Spain; Nephrology Department, Vall d’Hebron University Hospital, Vall d’Hebron Research Institute (VHIR), Vall d’Hebron Barcelona Hospital Campus, Autonomous University of Barcelona, Barcelona, Spain; Nephrology Department, Vall d’Hebron University Hospital, Vall d’Hebron Research Institute (VHIR), Vall d’Hebron Barcelona Hospital Campus, Autonomous University of Barcelona, Barcelona, Spain; Nephrology Department, Vall d’Hebron University Hospital, Vall d’Hebron Research Institute (VHIR), Vall d’Hebron Barcelona Hospital Campus, Autonomous University of Barcelona, Barcelona, Spain; Nephrology Department, Vall d’Hebron University Hospital, Vall d’Hebron Research Institute (VHIR), Vall d’Hebron Barcelona Hospital Campus, Autonomous University of Barcelona, Barcelona, Spain; Nephrology Department, Vall d’Hebron University Hospital, Vall d’Hebron Research Institute (VHIR), Vall d’Hebron Barcelona Hospital Campus, Autonomous University of Barcelona, Barcelona, Spain; Nephrology Department, Vall d’Hebron University Hospital, Vall d’Hebron Research Institute (VHIR), Vall d’Hebron Barcelona Hospital Campus, Autonomous University of Barcelona, Barcelona, Spain; Nephrology Department, Vall d’Hebron University Hospital, Vall d’Hebron Research Institute (VHIR), Vall d’Hebron Barcelona Hospital Campus, Autonomous University of Barcelona, Barcelona, Spain; Nephrology Department, Vall d’Hebron University Hospital, Vall d’Hebron Research Institute (VHIR), Vall d’Hebron Barcelona Hospital Campus, Autonomous University of Barcelona, Barcelona, Spain; Nephrology Department, Vall d’Hebron University Hospital, Vall d’Hebron Research Institute (VHIR), Vall d’Hebron Barcelona Hospital Campus, Autonomous University of Barcelona, Barcelona, Spain; Nephrology Department, Vall d’Hebron University Hospital, Vall d’Hebron Research Institute (VHIR), Vall d’Hebron Barcelona Hospital Campus, Autonomous University of Barcelona, Barcelona, Spain; Catalan Transplantation Organization, Barcelona, Spain; Nephrology Department, Vall d’Hebron University Hospital, Vall d’Hebron Research Institute (VHIR), Vall d’Hebron Barcelona Hospital Campus, Autonomous University of Barcelona, Barcelona, Spain; Nephrology Department, Vall d’Hebron University Hospital, Vall d’Hebron Research Institute (VHIR), Vall d’Hebron Barcelona Hospital Campus, Autonomous University of Barcelona, Barcelona, Spain; Nephrology Department, Vall d’Hebron University Hospital, Vall d’Hebron Research Institute (VHIR), Vall d’Hebron Barcelona Hospital Campus, Autonomous University of Barcelona, Barcelona, Spain

**Keywords:** dialysis modality, end-stage kidney disease, hemodialysis, octogenarians, peritoneal dialysis, propensity score matching, survival analysis

## Abstract

**Background:**

Evidence guiding dialysis modality decisions in very elderly patients is limited, and real-world outcomes remain poorly characterized. This study evaluates long-term survival in octogenarians initiating kidney replacement therapy (KRT) by dialysis modality.

**Methods:**

We used data from the Catalan Renal Registry to identify all patients aged ≥80 years who started dialysis between 2000 and 2022. Patients were classified according to initial dialysis modality [hemodialysis (HD) vs peritoneal dialysis (PD)]. A 1:3 propensity score-matched analysis was performed. Transition from PD to HD was modeled as a time-dependent covariate in Cox proportional hazards models to account for modality switching and minimize immortal-time bias.

**Results:**

Among 4205 octogenarians, 239 initiated PD, 3966 initiated HD. After matching, 213 PD patients were compared to 637 HD controls. Five-year survival did not differ significantly by initial modality after adjustment. However, switching from PD to HD was independently associated with increased mortality (HR 1.46; 95% CI 1.02–2.08; *P* = .038), suggesting a potential association with clinical deterioration. PD without switching was not significantly associated with mortality (HR 1.19; *P* = .120; 95% CI 0.95–1.48). Older age and KRT initiation in 2000–07 were also associated with worse outcomes.

**Conclusions:**

In this large population-based cohort of octogenarians, initial dialysis modality was not associated with differences in survival after adjustment. However, transition from PD to HD was associated with higher mortality, underscoring the importance of dynamic monitoring and ongoing clinical reassessment in peritoneal dialysis.

## Graphical Abstract

**Graphical Abstract:**
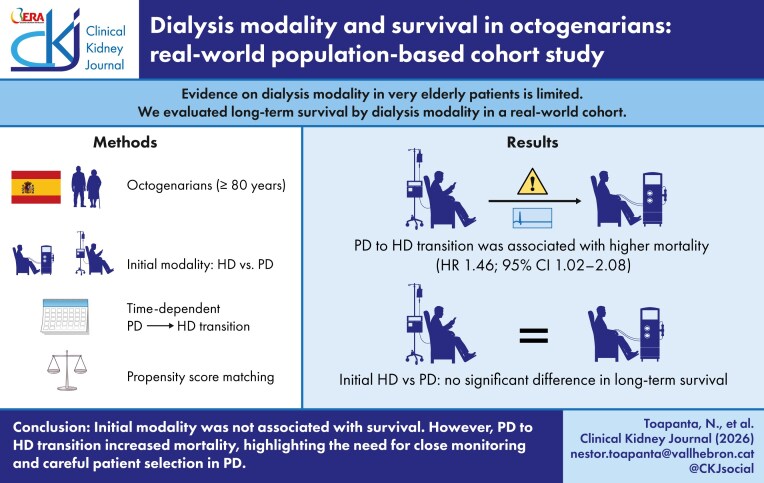

KEY LEARNING POINTS **What was known:**

- Octogenarians are a growing dialysis population, but robust, comparative survival data by dialysis modality in this age group remain scarce.
- PD is commonly preferred in elderly patients due to better hemodynamic tolerance and autonomy, though outcomes are inconsistent across studies.
- Prior studies rarely used time-dependent analyses to address modality switches, risking biased survival estimates.

**This study adds:**

- In a large matched cohort, 5-year survival did not differ significantly between HD and PD when accounting for modality transitions and baseline confounders.
- Switching from PD to HD was independently associated with increased mortality, likely reflecting clinical deterioration or technique failure.
- These results challenge a presumed survival benefit of PD in the very elderly.

**Potential impact:**

- PD remains a valid initial choice in octogenarians, but should include careful planning for potential failure and transition.
- Clinicians must evaluate long-term PD sustainability alongside clinical and social factors when recommending dialysis modality.
- Structured follow-up and early intervention may reduce excess mortality from unplanned PD-to-HD transitions in elderly patients.

## INTRODUCTION

The global increase in life expectancy and improved management of chronic conditions have led to a growing number of octogenarians initiating kidney replacement therapy (KRT). Individuals aged ≥80 years now represent a significant and expanding subset of incident dialysis patients across healthcare systems worldwide [1–3]. Their care presents distinct clinical and ethical challenges, as advanced age is often accompanied by frailty, multimorbidity, cognitive impairment, and limited life expectancy [4–6]. Despite this demographic shift, currently the optimal dialysis modality for this vulnerable group remains uncertain, and robust survival data are limited.

Peritoneal dialysis (PD) has long been considered an attractive option in selected elderly patients, due to its preservation of hemodynamic stability, avoidance of vascular access, and potential to support greater autonomy, particularly when adequate caregiver assistance is available [7–9, 10]. Some observational studies have suggested that PD may offer comparable or even superior short-term survival to hemodialysis (HD) during the first 2 years of treatment [11]. Moreover, the potential for improved quality of life, reduced treatment burden, and fewer hospital visits has led many centers to prioritize PD in older adults with appropriate social and functional conditions [7, 11, 12].

While HD remains the most widely used modality worldwide, in elderly patients likely reflecting greater physician familiarity and perceived predictability in metabolic and clinical management it carries significant risks [13–15]. In this population, HD is frequently associated with intradialytic hypotension, cardiovascular stress, vascular access complications, and repeated hospitalizations, all of which may negatively affect both survival and quality of life [16, 17].

The long-term impact of initial dialysis modality and especially the clinical implications of transitioning from PD to HD remains poorly defined in octogenarians. Hence, we aimed to compare long-term survival according to initial dialysis modality, and to assess the prognostic impact of subsequent transition from PD to HD using a propensity score–matched design and analyses correcting for immortal time bias.

## MATERIALS AND METHODS

### Study design and population

This was a retrospective observational cohort study based on data from the Catalan Renal Registry (Registre de Malalts Renals de Catalunya, RMRC). We included all patients aged ≥80 years who initiated dialysis between 1 January 2000, and 31 December 2022. Patients were excluded if they had previously received KRT outside Catalonia, experienced early recovery of kidney function, or switched from HD to PD within the first 90 days after dialysis initiation. Early HD-to-PD switches were excluded because these cases typically correspond to urgent-start HD followed by planned PD initiation once the patient becomes clinically stable, rather than representing a true modality transition.

Ethical approval was obtained from the institutional review board of the RMRC (approved by the Catalan Government; DOGC 402, 27 January 1984), and all patient data were pseudonymized before analysis.

### Study groups

Patients were classified according to initial dialysis modality at day 0 (KRT start) into two groups: HD and PD. This binary classification was used for propensity score matching, reflecting the clinical decision at initiation.

Among patients initiating PD, subsequent transition to HD was handled as a time-varying event (technique switch) rather than defining a third baseline group, in order to avoid immortal time bias. Accordingly, PD patients contributed person-time to the PD state until the date of transfer to HD and to the HD state, thereafter, as modeled in time-dependent analyses.

### Data collection

Collected variables included demographics (age, sex), comorbidities (ischemic heart disease, diabetes mellitus, malignancy, chronic pulmonary disease), functional status (independent, partially dependent, completely dependent), social factors (living situation, literacy, support), and dialysis period. Functional autonomy and social circumstances were also recorded.

### Outcomes and follow-up

The primary outcome was overall survival, defined as the time from dialysis initiation to death or end of follow-up. Secondary outcomes included transfer between modalities and post-transfer survival. Patients were followed until death, loss to follow-up, kidney transplantation, or 31 December 2022.

Kidney transplantation was treated as a censoring event in survival analyses, as the primary objective of the study was to evaluate mortality among patients receiving dialysis therapy. Transition from PD to HD was not treated as a competing event but rather modeled as a time-dependent covariate in Cox regression models, allowing patients to contribute person-time to PD before transfer and to HD thereafter.

Patients who experienced early recovery of kidney function were excluded because these cases were considered more consistent with acute or acute-on-chronic kidney dysfunction rather than established chronic KRT. Explicit transition toward conservative management after dialysis initiation was very uncommon in the registry cohort (*n* = 4). Given the very small number of these cases, they were not analyzed as a separate subgroup and were excluded from the primary analyses.

### Statistical analysis

Variables were summarized as mean ± standard deviation (SD), median and interquartile range (IQR), or frequencies, as appropriate. Continuous variables were compared using Student’s *t*-test or the Mann–Whitney *U* test, depending on distribution. Categorical variables were compared using chi-square or Fisher’s exact test, as appropriate.

Kaplan–Meier survival curves were used to describe unadjusted survival across dialysis modalities at 1, 3, and 5 years, and differences between groups were assessed using the log-rank test. Because modality transitions may occur during follow-up in dialysis populations, cumulative incidence functions were also estimated to explore the occurrence of competing events, including modality transfer and kidney transplantation. The primary analytical approach for estimating mortality risk was based on Cox proportional hazards models incorporating modality transition as a time-dependent covariate. A *P*-value <.05 was considered statistically significant.

To minimize confounding due to baseline differences between dialysis modalities, a 1:3 propensity score matching (PSM) was performed using age, sex, comorbidities, functional status, dialysis initiation period, region of residence (health region) and social variables. Covariate balance after propensity score matching was assessed using standardized mean differences (SMD), with values <0.1 considered indicative of adequate balance between groups. Covariate balance before and after matching is illustrated in Supplementary Fig. S1, and the distribution of propensity scores in both groups before and after matching is shown in Supplementary Fig. S2.

The primary analyses were performed in the propensity score–matched cohort. Survival analyses were conducted using Cox proportional hazards models to estimate hazard ratios (HRs) for all-cause mortality. The Cox proportional hazards models were fitted in the propensity score–matched cohort and included dialysis modality as a time-dependent covariate. The multivariable models adjusted for age, sex, dialysis initiation period, presentation of kidney failure, functional status, diabetes mellitus, and cardiovascular comorbidity.

Among patients initiating PD, transfer to HD was modeled as a time-dependent covariate, allowing patients to contribute person-time to each modality and preventing immortal time bias. Kidney transplantation was treated as a censoring event in all survival analyses. Patients who recovered kidney function after dialysis initiation were excluded from the analytical cohort.

To evaluate the potential impact of unmeasured confounding on the estimated associations between dialysis modality and mortality, we performed a sensitivity analysis using the E-value approach [18]. The E-value quantifies the minimum strength of association that an unmeasured confounder would need to have with both the exposure and the outcome, beyond the measured covariates, to fully explain the observed association.

Missing data were assessed for all variables included in the propensity score model and multivariable analyses. Patients with missing values in the covariates used for propensity score matching were excluded from the matching procedure. Survival analyses comparing patients with and without missing values did not show significant differences (data not shown), suggesting that the exclusion of these patients did not introduce relevant bias.

Overall, 525 HD patients (13.2%) and 25 PD patients (10.5%) had missing values in at least one matching covariate and were excluded from the matched analysis. Importantly, survival analyses comparing patients with and without missing values did not show significant differences between groups, suggesting that exclusion of incomplete cases was unlikely to introduce major bias. Given the relatively limited proportion of missing data and the absence of relevant survival differences, a complete-case approach was considered appropriate and multiple imputation was not performed.

## RESULTS

### Cohort description and patient distribution

Between 2000 and 2022, a total of 24 652 patients initiated KRT with dialysis in Catalonia. Of these, 4256 (17.2%) were aged 80 years or older. Patients were excluded if they had previously received KRT outside the region, experience early recovery of kidney function, or switched from HD to PD within the first 90 days after dialysis initiation. Early HD-to-PD switches (*n* = 9) were excluded because these cases typically correspond to urgent-start HD followed by planned PD initiation once the patient becomes clinically stable, rather than representing a true initial modality transition.

**Figure 1:**
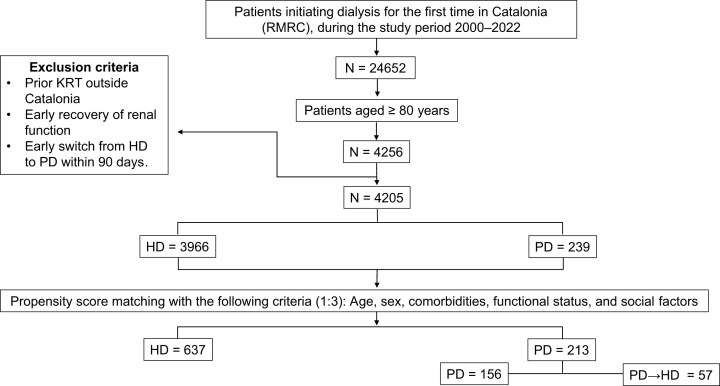
Flow chart of the study population. Patients aged ≥80 years initiating kidney replacement therapy (KRT) with dialysis in Catalonia between 2000 and 2022 were identified from the Catalan Renal Registry (RMRC). Patients with prior KRT outside Catalonia, early recovery of kidney function, or switch from hemodialysis (HD) to peritoneal dialysis (PD) within 90 days were excluded.

After applying these criteria, the final cohort included 4205 patients. Among them, 3966 (94.3%) initiated and remained on HD, 176 (4.2%) were treated exclusively with PD, and 63 (1.5%) started on PD but later transitioned to HD (PD→HD) (Fig. 1).

### Baseline characteristics (unmatched cohort)

In the unmatched cohort, patients in the PD group showed higher levels of independence, a lower burden of cardiovascular comorbidities, and more frequently lived with a partner. Median follow-up time was similar across groups: HD 3 years (IQR 1–6), PD 3 years (IQR 1–6) (Table 1).

**Table 1:** Baseline characteristics (unmatched cohort).

Variable	HD (*n* = 3966)	PD (*n* = 239)	*P*
Period			
2000–07, %	96.6	3.3	<.001
2008–15, %	94.8	5.1	
2016–22, %	92.4	7.5	
Mean age (SD), years	83.2(2.7)	83.1(2.6)	.088
Sex (male/female), %	61.3/38.7	67.4/32.6	.042
Etiology of CKD			
Unknown, %	41.0	43.3	.577
Glomerular disease, %	7.8	5.2	
Tubulointerstitial disease, %	6.2	7.7	
Hereditary/familial disease, %	3.1	3.0	
Hypertension/vascular disease, %	20.6	22.7	
Diabetes, %	17.9	16.3	
Other systemic diseases, %	3.4	4.0	
Median follow-up (years, IQR)	3 (1–6)	3 (1–6)	.247
Living situation			
Lives with partner, %	84.7	89.9	.073
Lives alone, %	7.4	6.9	
Lives alone with external support, %	3.3	1.8	
Lives in nursing home, %	4.6	1.4	
Education level			
Illiterate, %	3.5	0.5	.001
Primary, %	72.5	65.7	
Secondary, %	15.8	19.9	
High school, %	5.6	6.9	
University, %	2.7	6.9	
Degree of functional autonomy			
Independent, %	15.9	24.3	.001
Partially dependent, %	82.3	75.7	
Completely dependent, %	1.8	0.0	
Comorbidity			
Ischemic heart disease, %	25.2	28.5	.916
Cardiomyopathy or heart failure, %	38.5	27.2	.001
Conduction disorders, %	30.4	31.8	.868
Cardiac surgery, %	5.5	7.4	.305
Cerebrovascular disease, %	15.4	8.1	.028
Vascular disease, %	22.1	12.8	.001
COPD, %	22.8	15.3	.001
Tuberculosis, %	2.4	1.2	.261
Malignant tumors, %	34.5	33.3	.532
Diabetes mellitus, %	39.0	36.3	.279
Arthropathy, %	35.7	34.3	.153
Liver disease, %	4.2	4.1	.761
Esophageal, stomach or duodenal disease, %	10.7	6.4	.136
Intestinal disease, %	10.1	4.1	.001
Hypertension, %	89.4	87.3	.552
Irreversible severe visual deficit, %	4.4	5.2	.853
Psychiatric disorders, %	4.4	3.6	.204
≥1 cardiovascular condition (5 diseases), %	65.7	58.5	.026
Waiting list status			
Included, %	1.0	2.4	.157
Pending studies, %	15.5	12.8	
Definitively excluded, %	83.5	85.0	

Notes: HD, hemodialysis; PD, peritoneal dialysis; SD, standard deviation; CKD, chronic kidney disease; IQR, interquartile range; COPD, chronic obstructive pulmonary disease.

Missingness across variables included in the primary analyses was generally low to moderate and did not require imputation.

**Figure 2:**
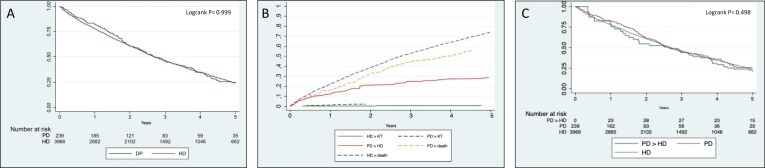
Unadjusted survival and descriptive cumulative incidence curves at 5 years in the unmatched cohort. (A) Kaplan–Meier survival according to initial dialysis modality. (B) Descriptive cumulative incidence curves showing death, kidney transplantation, and transition from peritoneal dialysis (PD) to hemodialysis (HD). Transition from PD to HD is displayed descriptively and was modeled as a time-dependent covariate in the survival analyses rather than as a competing risk. (C) Kaplan–Meier survival according to observed modality trajectory (HD, PD, and PD→HD).

Unadjusted Kaplan–Meier survival analysis over 5 years showed no significant difference in survival between HD and PD (Log-rank *P* = .999; Fig. 2A). However, competing risk analysis revealed that transition to HD acted as a competing event for death in the PD group, potentially underestimating mortality if not accounted for (Fig. 2B). In contrast, patients in the HD group, who did not experience modality transitions, showed a higher cumulative incidence of death, consistent with the absence of competing risks. When dialysis trajectories were summarized descriptively as HD without switching, PD without switching, and PD with subsequent transfer to HD (PD→HD), 5-year survival differences remained non-significant (log-rank *P* = .498; Fig. 2C). Importantly, this trajectory-based comparison was descriptive, whereas the primary adjusted analysis modeled transfer from PD to HD as a time-dependent covariate.

### Propensity score–matched cohort

**Figure 3:**
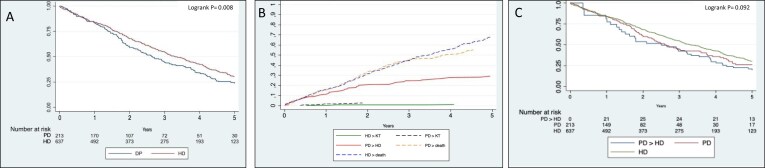
Survival and descriptive cumulative incidence curves at 5 years in the propensity score–matched cohort. (A) Kaplan–Meier survival according to initial dialysis modality after propensity score matching. (B) Descriptive cumulative incidence curves showing death, kidney transplantation, and transition from peritoneal dialysis (PD) to hemodialysis (HD). Transition from PD to HD is displayed descriptively and was modeled as a time-dependent covariate in the survival analyses rather than as a competing risk. (C) Kaplan–Meier survival according to observed modality trajectory (HD, PD, and PD→HD) in the matched cohort.

A 1:3 propensity score matching was applied based on age, sex, comorbidities, functional status, and social variables. The resulting matched cohort included 637 HD patients and 213 PD patients. After matching, baseline characteristics across the groups were well balanced. (Tables 2 and 3). In the matched cohort, Kaplan–Meier survival curves showed a significantly lower survival probability in patients initiating dialysis with PD compared to those on HD (log-rank *P* = .008; Fig. 3A).

**Table 2:** Baseline characteristics (matched cohort).

Variable	HD (*n* = 637)	PD (*n* = 213)	*P*
Period			
2000–07, %	77.7	22.2	.602
2008–15, %	86.4	28.3	
2016–22, %	73.7	26.2	
Mean age (SD), years	82.9(2.5)	83.0(2.6)	.635
Sex (male/female), %	64.4/35.6	65.7/34.3	.718
Etiology of CKD			
Unknown, %	41.0	41.3	.415
Glomerular disease, %	7.7	5.6	
Tubulointerstitial disease, %	6.6	8.5	
Hereditary/familial disease, %	5.2	2.8	
Hypertension/vascular disease, %	19.0	22.5	
Diabetes, %	17.3	17.8	
Other systemic diseases, %	3.1	1.4	
Median follow-up (years, IQR)	3.2 (1.3–7.0)	3 (1.0–6.0)	.425
Living situation			
Lives with partner, %	88.9	89.7	.895
Lives alone, %	8.0	7.0	
Lives alone with external support, %	2.2	1.9	
Lives in nursing home, %	0.9	1.4	
Education Level			
Illiterate, %	3.7	0.5	.002
Primary, %	74.0	66.5	
Secondary, %	13.9	18.9	
High school, %	5.6	7.1	
University, %	2.9	7.1	
Degree of functional autonomy			
Independent, %	23.7	23.5	.945
Partially dependent, %	76.3	76.5	
Comorbidity			
Ischemic heart disease, %	22.8	23.2	.186
Cardiomyopathy or heart failure, %	30.9	28.2	.448
Conduction disorders, %	24.8	31.9	.042
Cardiac surgery, %	5.8	7.5	.383
Cerebrovascular disease, %	13.3	10.3	.251
Vascular disease, %	18.8	13.2	.058
COPD, %	14.4	15.0	.835
Tuberculosis, %	1.6	1.4	.868
Malignant tumors, %	31.4	32.4	.786
Diabetes mellitus, %	34.9	36.2	.731
Arthropathy, %	36.1	33.3	.464
Liver disease, %	4.1	4.2	.927
Esophageal, stomach or duodenal disease, %	11.0	8.5	.292
Intestinal disease, %	8.5	3.3	.011
Hypertension, %	93.1	93.4	.863
Irreversible severe visual deficit, %	5.4	4.7	.711
Psychiatric disorders, %	2.9	2.9	.992
≥1 cardiovascular condition (5 diseases), %	57.5	60.1	.499
Waiting list status			
Included, %	1.3	2.3	.367
Pending studies, %	15.3	12.7	
Definitively excluded, %	83.5	85.0	

Notes: HD, hemodialysis; PD, peritoneal dialysis; SD, standard deviation; CKD, chronic kidney disease; IQR, interquartile range; COPD, chronic obstructive pulmonary disease.

**Table 3:** Clinical characteristics of PD patients with and without switch to HD (matched cohort).

Variable	PD (*n* = 156)	PD → HD (*n* = 57)	*P*
Period			
2000–07, %	82.3	17.6	.253
2008–15, %	67.1	32.8	
2016–22, %	74.1	25.8	
Mean age (SD), years	83.2(2.7)	82.3(2.3)	.035
Sex (male/female), %	62.2/37.8	75.4/24.6	.071
Etiology of CKD			
Unknown, %	41.0	42.1	.942
Glomerular disease, %	6.4	3.5	
Tubulointerstitial disease, %	9.0	7.0	
Hereditary/familial disease, %	2.6	3.5	
Hypertension/vascular disease, %	21.8	24.6	
Diabetes, %	17.3	19.3	
Other systemic diseases, %	1.9	0.0	
Median follow-up (years, IQR)	3 (1–6)	2.6 (1–5)	.684
Living situation			
Lives with partner, %	90.4	87.7	.878
Lives alone, %	6.4	8.8	
Lives alone with external support%	1.9	1.8	
Lives in nursing home, %	1.3	1.8	
Education Level			
Illiterate, %	0.7	0.0	.908
Primary, %	67.7	63.2	
Secondary, %	18.1	21.1	
High school, %	6.5	8.8	
University, %	7.1	7.0	
Degree of functional autonomy			
Independent, %	23.1	24.6	.821
Partially dependent, %	76.9	75.4	
Comorbidity			
Ischemic heart disease, %	30.1	19.3	.116
Cardiomyopathy or heart failure, %	28.2	28.1	.985
Conduction disorders, %	32.7	29.8	.691
Cardiac surgery, %	7.6	7.4	.968
Cerebrovascular disease, %	8.3	15.8	.113
Vascular disease, %	13.5	12.3	.821
COPD, %	16.7	10.5	.267
Tuberculosis, %	1.3	1.8	.796
Malignant tumors, %	32.7	31.6	.878
Diabetes mellitus, %	37.2	33.3	.605
Arthropathy, %	37.2	22.8	.049
Liver disease, %	4.5	3.5	.753
Esophageal, stomach, or duodenal disease, %	7.1	12.3	.224
Intestinal disease, %	4.5	0.0	.104
Hypertension, %	93.0	94.7	.641
Irreversible severe visual deficit, %	5.1	3.5	.621
Psychiatric disorders, %	4.0	0.0	.127
≥1 cardiovascular condition (5 diseases), %	60.3	59.7	.936
Waiting list status			
Included, %	2.6	1.5	.385
Pending studies, %	10.9	14.6	
Definitively excluded, %	86.5	83.9	

Notes: HD, hemodialysis; PD, peritoneal dialysis; SD, standard deviation; CKD, chronic kidney disease; IQR, interquartile range; COPD, chronic obstructive pulmonary disease.

Competing-risk analyses showed that transfer from PD to HD occurred before death in a proportion of PD patients and therefore acted as a competing event. As a result, estimates of death based on standard survival methods that do not account for modality changes may be biased (Fig. 3B).

When analyzing survival by modality group (HD, PD, PD→HD), no statistically significant differences were observed at 5 years (log-rank *P* = .092), although patients who switched from PD to HD appeared to have slightly lower survival compared to the other groups (Fig. 3C).

### Multivariable cox regression

A Cox proportional hazards model incorporating modality switch as a time-dependent covariate revealed the following: Transition from PD to HD was independently associated with increased mortality (HR 1.46; 95% CI: 1.02–2.08; *P* = .038). PD without switch was not significantly associated with mortality compared with HD (HR 1.19; 95% CI: 0.95–1.48; *P* = .120). Older age and KRT initiation between 2000 and 2007 were also independently associated with increased mortality (Table 4 ).

**Table 4:** Cox proportional Hazards model: 5-year mortality risk (matched cohort).

Variable	Hazard ratio	Std. error	z	*P*-value	95% CI
PD → HD	1.46	0.2651	2.08	.038	1.02–2.08
PD no switch	1.19	0.1340	1.56	.120	0.95–1.48
Period 2000–07	1.37	0.1635	2.68	.007	1.08–1.73
Period 2008–15	1.14	0.1167	1.34	.180	0.93–1.39
Male (vs. female)	1.16	0.1095	1.57	.117	0.96–1.40
Age (per year)	1.06	0.0185	3.70	<.001	1.03–1.10

**Table 5:** Reasons for transition from peritoneal dialysis to hemodialysis.

Reason for transition	*n*	%
Loss of peritoneal membrane function	17	30.4
Severe intercurrent illness/major complication	17	30.4
Peritoneal infection	13	23.2
Patient choice	9	16.1
Unknown	7	4.4

Sensitivity analyses using the E-value approach were performed to assess the potential impact of unmeasured confounding. For the comparison between patients remaining on PD and those treated exclusively with HD, the observed hazard ratio would require an unmeasured confounder associated with both exposure and mortality by a risk ratio of at least 1.72 to fully explain the observed association. For the comparison between patients who transitioned from PD to HD and those treated exclusively with HD, the corresponding value was 2.02. Graphical representations of the E-value sensitivity analyses are provided in Supplementary Fig. S3.

### Reasons for technique transition (PD→HD)

Among the 63 patients who transitioned from PD to HD, the timing of modality switch was highly variable, with a median time on PD of 461 days (IQR 144–944). Reasons for transfer were heterogeneous. Among the 56 patients with available information on the cause of transition, the most frequent reasons were loss of peritoneal membrane function (17 patients, 30.4%), intercurrent severe comorbid conditions (17 patients, 30.4%), followed by PD–related infections, including peritonitis or tunnel infections (13 patients, 23.2%), and patient choice (9 patients, 16.1%). The cause of transfer was unavailable in seven cases (Table 5).

## DISCUSSION

In this large real-world cohort of octogenarians initiating KRT, we observed that initial dialysis modality was not independently associated with differences in long-term survival after adjustment for baseline characteristics. However, transition from PD to HD was independently associated with increased mortality, underscoring the clinical relevance of modality transitions in this population.

A key strength of our study is the explicit modeling of dialysis modality as a time-dependent exposure. By allowing patients to contribute person-time to each modality, this approach minimizes immortal time bias and provides a more accurate estimation of the association between dialysis modality and survival [19, 20]. This is particularly relevant in elderly populations, where modality transitions are frequent and often reflect evolving clinical conditions rather than fixed treatment strategies.

In unadjusted analyses, survival appeared similar across modalities, while in the matched cohort PD initiation was associated with lower survival in Kaplan–Meier analysis. However, this apparent difference should be interpreted with caution, as Kaplan–Meier methods do not account for modality transitions over time. When modality changes were appropriately incorporated using time-dependent Cox models, the association between PD without switching and mortality was attenuated and no longer statistically significant. These findings suggest that apparent survival differences across modalities may be largely driven by baseline selection and by analytical approaches that do not adequately account for treatment changes over time [19, 21]. Our results therefore challenge the assumption of a clear survival advantage of PD in very elderly patients and align with previous studies showing neutral or context-dependent effects once appropriate adjustment methods are applied [21].

The most consistent and clinically relevant finding of our study was the increased mortality observed in patients who transitioned from PD to HD. Importantly, this association should not be interpreted as a direct causal effect of the modality switch itself. Rather, it likely reflects underlying clinical deterioration, complications, or progressive loss of suitability for PD [22, 23]. Nevertheless, we cannot exclude that the transition process itself may contribute to adverse outcomes through hospitalization, vascular access procedures, hemodynamic stress, inflammatory burden, or physiological instability in frail patients. The observational nature of the study precludes distinguishing whether modality transition acts primarily as a marker of vulnerability, a mediator of risk, or both. The heterogeneity in reasons for transition observed in our cohort including peritoneal membrane failure, intercurrent illness, infections, and patient-related factors, supports this interpretation, and highlights the complexity of treatment trajectories in this population.

Importantly, these clinical triggers for transition such as loss of peritoneal membrane function, acute intercurrent illness, or infection represent well-recognized pathways of clinical instability in elderly patients on PD. This reinforces that modality transition is typically a consequence of evolving clinical vulnerability rather than a predictable or preventable event at dialysis initiation.

From a clinical perspective, these findings emphasize the need to move beyond a static view of dialysis modality choice. In octogenarians, modality selection should be considered as part of a dynamic care pathway that requires continuous reassessment. PD remains an appropriate initial option in selected patients, particularly when supported by adequate social and healthcare resources [7, 10]. In clinical practice, PD in octogenarians may be particularly suitable for patients with preserved autonomy, stable clinical status, adequate cognitive function, supportive home environments, or access to assisted PD programs. However, the impact of caregiver-assisted or nurse-assisted PD on long-term outcomes in this population could not be evaluated in our registry and deserves further investigation.

However, the long-term sustainability of PD appears to be a key determinant of outcomes. In this context, close monitoring and early identification of technique failure are essential, and proactive planning for potential transition to HD may help mitigate the risks associated with late or unplanned switches [22, 24].

The observed outcomes in HD patients may partly reflect the benefits of structured in-center care, including frequent clinical monitoring and timely management of complications [17, 25]. However, this potential advantage is likely dependent on healthcare system organization and resource availability and should not be generalized without considering local context.

Our study has several strengths. It includes a large, population-based cohort covering more than two decades, with comprehensive capture of incident dialysis patients aged ≥80 years. The use of propensity score matching achieved good balance in measured baseline characteristics, and the incorporation of time-dependent analyses allowed for a more accurate representation of real-world dialysis trajectories. Additionally, the E-value analysis suggests that a moderately strong unmeasured confounder would be required to fully explain the observed associations, supporting the robustness of our findings [19, 21]. Nevertheless, E-values should be interpreted cautiously, as they estimate the strength of a hypothetical single unmeasured confounder and do not account for the cumulative effect of multiple interrelated factors. In elderly dialysis populations, frailty, cognitive impairment, nutritional status, caregiver availability, and functional dependency may jointly influence both modality selection and mortality risk. Although the magnitude of unmeasured confounding required to fully explain our findings appears relatively high, residual confounding cannot be completely excluded.

Several limitations should be acknowledged. As an observational study, residual confounding cannot be excluded, particularly from variables not captured in the registry such as frailty, cognitive status, nutritional parameters, and adequacy of home support, all of which may influence both modality selection and outcomes [10, 26]. Although the proportion of missing data was low and a complete-case approach was used, missingness inherent to registry data may still introduce uncertainty.

The relatively small number of patients who transitioned from PD to HD limits the precision of estimates for this subgroup. In addition, we did not explicitly model the duration of PD prior to transfer, which may influence outcomes and should be addressed in future studies. The exclusion of early HD-to-PD switches, although methodologically justified, may also introduce a degree of selection bias.

An additional source of potential bias relates to the exclusion of patients who switched from HD to PD within the first 90 days. These patients often represent urgent-start dialysis cases with distinct clinical characteristics. Although this approach was intended to better capture true initial modality choice, it may introduce selection bias and should be considered when interpreting the results.

Additionally, although kidney transplantation was treated as a censoring event, other clinically relevant competing events such as withdrawal from dialysis or transition to conservative care were not explicitly modeled. This may influence survival estimates in this elderly population. In addition, conservative kidney management was not evaluated as a treatment strategy because the RMRC includes only patients who initiated dialysis therapy. Future studies comparing dialysis modalities with conservative management in octogenarians are needed, particularly incorporating quality of life, symptom burden, and frailty-related outcomes.

Finally, the lack of center-level data precluded adjustment for variability in clinical practice across dialysis units. This is particularly relevant for PD, where program organization, training, and support structures may vary substantially between centers and could influence both modality sustainability and patient outcomes.

In conclusion, in octogenarian patients initiating dialysis, initial modality was not independently associated with long-term survival after adjustment. In contrast, transition from PD to HD was associated with increased mortality, likely reflecting underlying clinical deterioration rather than a direct effect of the modality change. These findings support a dynamic, individualized and patient-centered approach to dialysis modality selection, integrating not only survival expectations but also frailty, functional status, quality of life, and patient preferences.

These findings support a dynamic, individualized, and patient-centered approach to dialysis modality selection, integrating not only survival expectations but also frailty, functional status, quality of life, and patient preferences.

## Supplementary Material

sfag233_Supplemental_File

## Data Availability

The data underlying this article will be shared on reasonable request to the corresponding author.
